# Salt-Inducible Kinase 1 (SIK1) Is Induced by Gastrin and Inhibits Migration of Gastric Adenocarcinoma Cells

**DOI:** 10.1371/journal.pone.0112485

**Published:** 2014-11-10

**Authors:** Linn-Karina M. Selvik, Shalini Rao, Tonje S. Steigedal, Ildri Haltbakk, Kristine Misund, Torunn Bruland, Wenche S. Prestvik, Astrid Lægreid, Liv Thommesen

**Affiliations:** 1 Department of Cancer Research and Molecular Medicine, Norwegian University of Science and Technology (NTNU), Trondheim, Norway; 2 Department of Technology, Sør-Trøndelag University College, Trondheim, Norway; Rutgers University, United States of America

## Abstract

Salt-inducible kinase 1 (SIK1/Snf1lk) belongs to the AMP-activated protein kinase (AMPK) family of kinases, all of which play major roles in regulating metabolism and cell growth. Recent studies have shown that reduced levels of SIK1 are associated with poor outcome in cancers, and that this involves an invasive cellular phenotype with increased metastatic potential. However, the molecular mechanism(s) regulated by SIK1 in cancer cells is not well explored. The peptide hormone gastrin regulates cellular processes involved in oncogenesis, including proliferation, apoptosis, migration and invasion. The aim of this study was to examine the role of SIK1 in gastrin responsive adenocarcinoma cell lines AR42J, AGS-G_R_ and MKN45. We show that gastrin, known to signal through the Gq/G_11_-coupled CCK2 receptor, induces SIK1 expression in adenocarcinoma cells, and that transcriptional activation of SIK1 is negatively regulated by the Inducible cAMP early repressor (ICER). We demonstrate that gastrin-mediated signalling induces phosphorylation of Liver Kinase 1B (LKB1) Ser-428 and SIK1 Thr-182. Ectopic expression of SIK1 increases gastrin-induced phosphorylation of histone deacetylase 4 (HDAC4) and enhances gastrin-induced transcription of *c-fos* and CRE-, SRE-, AP1- and NF-κB-driven luciferase reporter plasmids. We also show that gastrin induces phosphorylation and nuclear export of HDACs. Next we find that siRNA mediated knockdown of SIK1 increases migration of the gastric adenocarcinoma cell line AGS-G_R_. Evidence provided here demonstrates that SIK1 is regulated by gastrin and influences gastrin elicited signalling in gastric adenocarcinoma cells. The results from the present study are relevant for the understanding of molecular mechanisms involved in gastric adenocarcinomas.

## Introduction

Gastrin is a regulatory peptide hormone which plays a crucial role in integration of exocrine and endocrine functions in the gastrointestinal tract. We and others have shown that gastrin regulates several important cellular processes in the gastric epithelium and in adenocarcinoma cells including proliferation [1], anti-apoptosis [2], [3], [4], migration [5] and invasion [6]. Functional genomics approaches have identified a range of new gastrin target genes [3], [4], [7], [8], [9]. By genome wide gene expression profiling we found that gastrin induces transcription of *Salt-inducible kinase 1* (*Sik1/Snf1lk*) in the pancreatic adenocarcinoma cell line AR42J [4]. SIK1 is a member of the AMP-activated protein kinase (AMPK) family. The AMPKs play major roles in regulating metabolism and cell growth [10], [11], [12]. Clinical studies have shown that reduced levels of SIK1 are associated with distal metastases and poor outcome in breast cancer, and SIK1 expression has been associated with a tumour suppressor function [13], [14], [15], [16]. Cheng and co-authors [16] have demonstrated that SIK1 links the tumour suppressor Liver Kinase 1B (LKB1) to p53-dependent suppression of metastasis and that SIK1 activated by LKB1 suppresses metastasis and invasion in a human mammary epithelial cell line [16]. The LKB1-AMPK pathway is also shown to serve as a metabolic checkpoint by arresting cell growth in conditions of low intracellular ATP levels [10].

Cell migration is important in normal and pathologic gastric epithelial function [6]. Since cell migration and invasion are characteristics of the progression of gastric cancer [6], and processes known to be regulated by gastrin, we wanted to address the role of SIK1 in gastrin-mediated responses. In the present study we show that gastrin induces transient SIK1 expression in pancreatic and gastric adenocarcinoma cells, and that the gastrin-induced *SIK1* expression is negatively regulated by Inducible cAMP early repressor (ICER). Ectopically expressed SIK1 increases gastrin-induced transcription of *c-fos* and CRE-, SRE-, AP1- and NF-κB-driven reporter plasmids. We also find that gastrin induces nuclear export of HDACs and ectopic SIK1 expression increases phosphorylation of class IIa HDAC4. Notable, the expression of *MMP-9* is almost abolished in cells with ectopic expression of SIK1, indicating that the effect of SIK1 may affect chromatin modifying events in different ways. Interestingly, we find that siRNA mediated knockdown of SIK1 enhances gastrin-induced migration of AGS-G_R_ cells. Collectively, this suggests a role of SIK1 in gastrin induced responses and suggest that SIK1 may act as tumour suppressor in gastric adenocarcinoma cells.

## Materials and Methods

### Cell lines

AR42J (rat pancreatic acinar cell derived; American Type Culture Collection (ATTC), Rockville, MD) were grown in DMEM with 4.5 g/l glucose (Invitrogen), 15% fetal bovine serum (FBS; Bio Whittaker, Lonza Belgium), 1 mM sodium pyruvate, 0.1 mg/ml L-glutamine (Invitrogen), 10 U/ml penicillin-streptomycin (Invitrogen) and 1 µg/ml fungizone (Invitrogen). AGS-G_R_ cells (human gastric adenocarcinoma, stably transfected with CCK2 receptor [17], [18]; gift from Prof. Andrea Varro, University of Liverpool, England) were maintained in Ham’s F-12 (Invitrogen, Carlsbad, CA) with 10% FBS, 10 U/ml penicillin-streptomycin and 2 µg/ml puromycin (Sigma-Aldrich, St. Louis, MO). MKN45 cells (human gastric adenocarcinoma; gift from Prof. Sue Watson, University of Nottingham) were grown in DMEM with 4.5 g/l glucose, 10% FCS, 10 U/ml penicillin-streptomycin and 1 µg/ml fungizone.

### Transient transfection and gastrin treatment of cells

AGS-G_R_ cells (5.0×10^5^/well) were seeded in six-well plates and transfected after 24 h with 2.5 µg plasmid and 12.5 µl Metafectene PRO transfection reagent (Biontex Laboratories GmbH, Martinsried, Germany) per well. 24 h after transfection, cells were serum starved for 24 h and treated with 5 nM gastrin as indicated in figures.

### qRT-PCR

Total RNA was isolated using the RNeasy Mini Kit (Qiagen, Germantown, MD). cDNA synthesis was performed with 1 µg total RNA in 20 µl reaction of Transcriptor First Strand cDNA Synthesis kit (Roche, Basel, Switzerland). After synthesis, cDNA was diluted 1∶2 with RNase-free water. qRT-PCR was performed with ABsolute QPCR SYBR Green Mix (Abgene Limited, Epsom, UK), 300 nM forward primer, 300 nM reverse primer, and cDNA equivalent to 62.5 ng total RNA in 25 µl reaction volume. qRT-PCR was performed in Stratagene's Mx3000P Real-Time PCR system: 15 min at 95°C, 40 thermal cycles of 15 s at 95°C, 20 s at 60°C, and 40 s at 72°C. Fold induction of gene expression of genes of interest was calculated by the ΔΔCt-method [19], where the expression levels were normalized to the level of GAPDH or B2M. The following primers were used: GAPDH, 5′-GAAGGTGAAGGTCGGAGTC-3' (sense) and 5′-GAAGATGGTGATGGGATTTC-3' (antisense); B2M, 5′- GAATTCACCCCCACTGAAAA-3' (sense) and 5′- AGCAAGCAAGCAGAATTTGG -3′ (antisense), ICER, 5′-TGGAACACTTTATGTTGAACTGTGG-3′ (sense) and 5′- CAAACTTCCGGGCGATGCAGCCATC -3′ (antisense); MMP-9, 5′-GACTTGGCAGTGGAGACTGCGGGCA-3′ (sense) and 5′-GACCCCACCCCTCCTTGACAGGCAA-3′ (antisense). RT^2^ qPCR Primer Assay for Human SNF1LK was purchased from SA Biosciences (Frederick, MD) (primer sequences not available).

### Plasmids and reagents

pIRES, pIRES-SIK1, pIRES-SIK1K56M, pIRES-SIK1S577A and pEGFPC-SIK1 were a kind gift from Dr. Hiroshi Takemori, National Institute of Biomedical Innovation, Japan. The plasmids pEF5/FRT/V5/GW-CAT, pEF5/FRT/ICER I and pEF5/FRT/ICER IIγ were constructed as previously described [20]. Empty control-vector was constructed by restriction cutting with BsrGI in the att sites of pEF5/FRT/V5-DEST. Gastrin-17 and cycloheximide were purchased from Sigma Chemical (St. Louis, MO). pSRE-luc and pc-fos-luc were a kind gift from Prof. Ugo Moens, University of Tromsø, Norway [21]. pCRE-luc containing four CRE somatostatin consensus promoter elements (TGACGTCA) and pNF-κB-luc, containing five copies of the NF-κB enhancer element (TGGGGACTTTCCGC), were obtained from Stratagene (La Jolla, CA). pAP1-luc containing four tandem copies of the AP1 consensus sequence (TGA(G/C)TCA) was obtained from Clontech (Palo Alto, CA). HDAC5 Flag plasmid was obtained from Add Gene Plasmid (# 13822).

### RNA interference experiments

Cells (3.0×10^5^/well) were seeded in six-well plates. After 24 h of cultivation, cells were transfected with 2.5 µg of siRNA (81 nM) and 12.5 µl of Metafectene PRO per well. 24 h after transfection, cells were serum starved for 24 h and then treated with 5 nM gastrin for 1 h. siSnf1lk (ID: 40587, 40679, 40762; a mix was used), Silencer Negative Control siRNA #1 and Silencer Negative Control siRNA #2 were purchased from Ambion (Carlsbad, CA). siRNA-All-ICER (Qiagen, Germantown, MD) were annealed at 20 mM in 1.0 ml siRNA suspension buffer (Qiagen). siRNA-All-ICER: 5′-CAUUAUGGCUGUAACUGGATT-3'. siRNA towards enhanced green fluorescent protein (EGFP) [22]; 5′-GCAAGCUGACCCUGAAGUUC-3'.

### Reporter gene assay

Cells (1.2×10^4^/well) were seeded in 96-well plates and transfected 24 h later. The cells were transfected with 84 ng plasmid, 1.68 ng (1∶50) phRL-null (Promega, Madison, WI) as internal control, and 0.42 µl Metafectene PRO per well for 24 h, serum starved for 24 h and then treated with gastrin for 4 h. Luciferase activity was measured with Wallac 1420 Victor3 plate reader (Perkin Elmer, Boston, MA) using the Dual Luciferase Reporter Assay System (Promega).

### Immunocytochemical staining and confocal microscopy

Cells (2.0×10^4^/well in 200 µl medium with 10% FBS) were seeded on Lab-Tek Chambered Coverglass with 8 wells (NUNC, Thermo Scientific, Rockford, IL) and transfected with pEGFPC-SIK1. After cultivation for 24 h, cells were serum starved for 24 h and then treated with 5 nM gastrin for 0–60 min. Living cells were examined by confocal microscopy. To examine endogenous CRTC2 and HDACs, cells were fixed (4% paraformaldehyde in PBS) for 10 min, washed (PBS x 2) and permeabilized (ice-cold MeOH) for 10 min on ice and washed (PBS x 2). Further, cells were blocked for 30 min (3% goat serum in PBS), incubated with the primary rabbit anti-human CRTC2 antibody (1∶500) or rabbit HDAC4/5/7 (Santa Cruz, CA) (1∶200) diluted in PBS with 1% goat serum for 30 min, washed 4×5 min (PBS), and incubated with secondary antibody (Alexa Fluor 488 or Alexa Fluor 546 (goat) anti-rabbit diluted 1∶1000 in PBS with 1% BSA) for 30 min. Cells were washed 4×5 min before DNA staining with Draq-5 (1∶1000) for 7 min, washed and stored at 4°C over night before confocal microscopy.

Confocal microscopy studies were performed with a Zeiss Axiovert 100-M inverted microscope equipped with an LSM 510 laser-scanning unit and a 1.4 numerical aperture ×63 Plan-Apochromat oil immersion objective. To examine living cells, the cell chamber was heated to 37°C using a Tempcontrol Digital 37-2 device (Warner Instruments). To minimize photobleaching, laser power was typically 20% under maximum, and the pinhole was set to 0.8–1.2. Multitracking was used for dual colour imaging. The Zeiss LSM Image browser version 4 was used for acquisition, and processing was completed using Adobe Illustrator CS5.

### Western blotting

Cells (5.0×10^5^/well) in six-well plates were harvested in 0.5 ml SDS-sample buffer and subjected to western blot analysis as described previously [23]. Binding of secondary antibodies was visualized by the Super Signal West Femto Maximum Sensitivity Substrate (Pierce, Rockford, IL) and Kodak Image Station 2000R (Kodak, Pittsburgh, PA). Band intensities were quantified using Adobe Photoshop Elements 8.0, and the intensities for the protein of interest were normalized to β actin intensities. The following antibodies were used: mouse monoclonal to beta actin (1∶2000; Abcam, Cambridge, MA), HRP-conjugated goat anti-mouse (1∶1000; Dako, Glostrup, Denmark), rabbit anti-human SNF1LK (1∶500, Abgent, San Diego, CA), HRP-conjugated goat anti-rabbit IgG (1∶1000; Cell Signaling, Beverly, MA), rabbit anti-human phospho-HDAC4 (Ser-632)/HDAC5 (Ser-498)/HDAC7 (Ser-486) antibody (1∶1000; Cell Signaling), rabbit anti-human phospho-LKB1 (Ser-428) (1∶1000; Cell Signaling) and Rabbit anti-phospho Thr-182 SIK1 (1∶500; generously provided by Dr H. Takemori (National Institute of Biomedical Innovation, Osaka, Japan)).

### Migration assay

The xCELLigence DP system (Roche Diagnostics GmbH, Germany) was used for measurement of migration. This system utilizes specialized culture plates containing gold electrode arrays beneath the bottom of individual wells. Cellular contact with the electrode surfaces increases the impedance across these gold arrays. This impedance value is measured by the DP system and reported in the dimensionless unit of cell index. Cells (3.0×10^5^/well) were seeded in six-well plates. After 24 h cultivation, cells were transfected with siRNA for 24 h, serum starved for 24 h, followed by reseeding (4.0×10^4^ cells/well) in CIM-Plate 16 (Roche). The lower chamber contained either 10% FBS or 10% FBS with 10 nM gastrin as attractant. Cell migration was monitored every 15 min on a RTCA DP instrument for 24 h. Data analysis was carried out using RTCA Software 1.2 supplied with the instrument.

### Statistical analyses

qRT-PCR and reporter gene experiments were performed with at least three biological replicates with three and six wells per condition per experiment, respectively. Results are shown as mean values ± SD for separate representative experiments, or as mean values ± SD/SEM for several experiments, as indicated in the figure legends. Student’s t-test was used to evaluate statistical significance where indicated.

## Results

### Gastrin induces SIK1 expression

**Figure 1:**
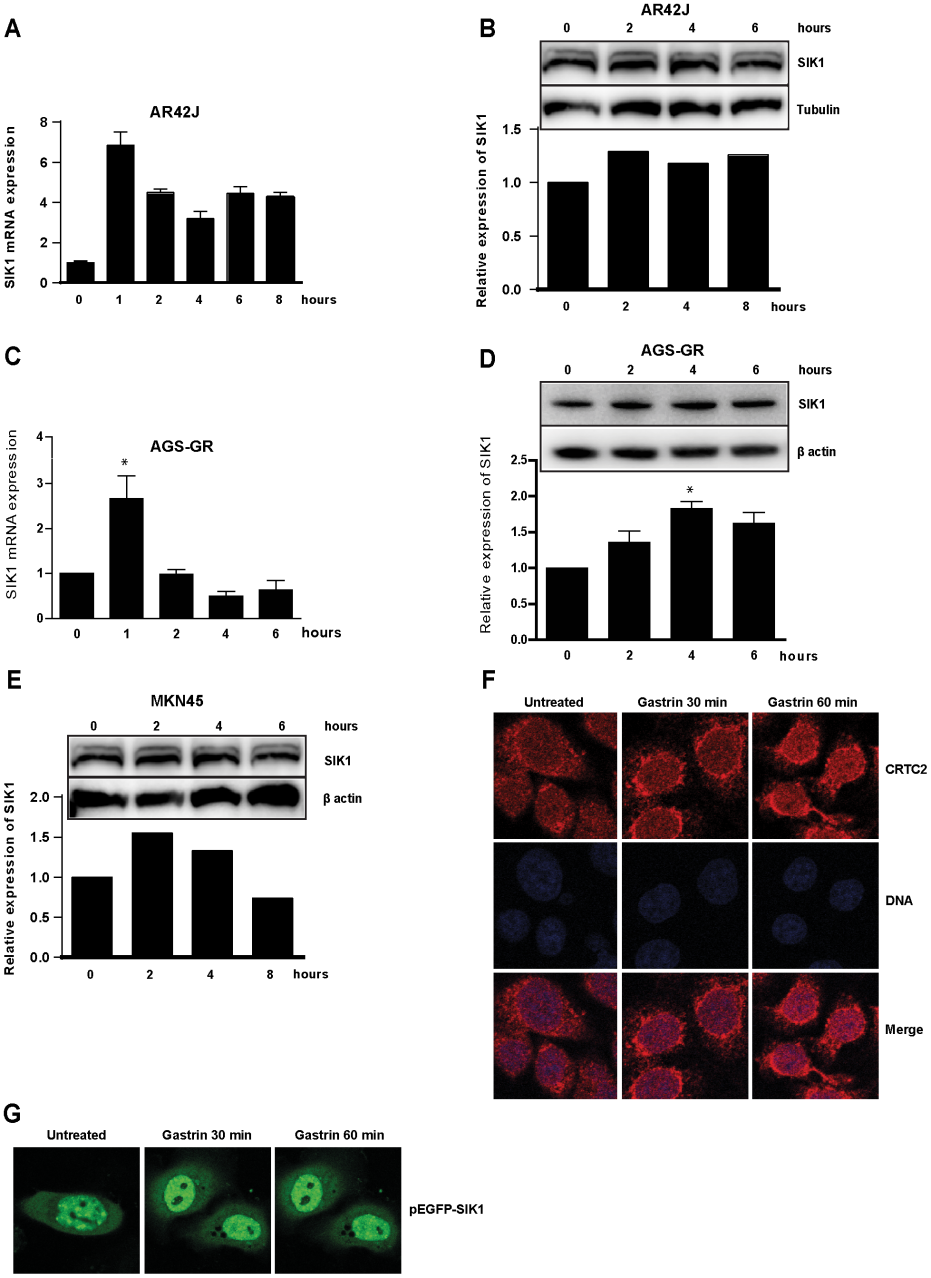
Gastrin-induced activation of SIK1. **A:** AR42J cells were treated with gastrin and mRNA levels measured by qRT-PCR. Mean expression level relative to untreated cells is shown. Results show one representative of three independent biological experiments; mean ± SD of three technical replicates. **B:** SIK1 Western blot of gastrin treated AR42J cells. A representative image is shown and quantified **C:** AGS-G_R_ cells were treated with gastrin and mRNA levels measured by qRT-PCR. Mean ± SEM of three independent biological experiments is shown. **D:** SIK1 Western blot of gastrin treated AGS-G_R_ cells. A representative image is shown and the SIK1 bands from two independent experiments were quantified; results shown are mean intensities ±SD. **E:** SIK1 Western blot of gastrin treated MKN45 cells. The SIK1 bands from a representative experiment were quantified. **F:** Intracellular localization of endogenous CRTC2 protein (Red; CRTC2, blue; Draq-5-stained DNA). G: Intracellular localization of SIK1 protein. AGS-G_R_ cells transfected with pEGFP-SIK1.

Microarray mRNA profiling of gastrin-treated pancreas derived adenocarcinoma cells (AR42J) demonstrated that gastrin transiently activates *Sik1* gene expression (Accession number: GSE32869). In the present study we further explored the role of SIK1 in the gastrin responsive cell lines AGS-G_R_, MKN45 and AR42J. Quantitative RT-PCR analyses confirmed that gastrin induces a rapid, transient increase of SIK1 mRNA in AR42J cells. The SIK1 mRNA increased approximately six fold upon gastrin treatment and we observed a small but reproducible enhancement of protein expression after 2 hrs (Figure 1A–B). In gastric adenocarcinoma AGS-G_R_ cells, the SIK1 mRNA was peaking at 1 h (Figure 1C). SIK1 protein accumulates 4–6 h after gastrin treatment (Figure 1D), and returns to basal level after 24 h (Figure S1A). We also found that gastrin treatment of MKN45 cells resulted in enhanced SIK1 protein expression (Figure 1E).

The *SIK1* promoter contains two cAMP responsive elements (CRE) and is transcriptionally regulated via the cAMP-CREB–CRTC2 pathway [24]. SIK1 protein itself regulates CRTC2 (CREB regulated transcription coactivator 2/TORC2) activity by phosphorylation of CRTC2 Ser-171 leading to its translocation to the cytoplasm with a subsequent inactivation of CREB [24], [25]. It is demonstrated that SIK1 plays a role in negative feedback of CREB/CRTC mediated transcription of steroidogenic enzymes [26]. To investigate whether SIK1 participates in regulation of the CRTC2-CREB axis also in gastric cancer cells, we examined the subcellular localization of CRTC2 protein upon gastrin treatment in AGS-G_R_ cells. We did not observe any evident translocation of CRTC2 proteins from nucleus to cytoplasm (Figure 1F), indicating that gastrin treatment does not affect shuttling of CRTC2 proteins and most likely does not modulate CREB activity via this pathway in AGS-G_R_. Since SIK1 has been reported to be transported out of the nucleus upon phosphorylation on Ser-577 [27], we also examined if gastrin treatment induced nucleo-cytoplasmic shuttling of SIK1 by use of EGFP-SIK1 fusion proteins. We found that EGFP-SIK1 displayed a punctual nuclear and cytoplasmic localization (Figure 1G), whereas no translocation was observed upon gastrin treatment.

### ICER is a negative regulator of gastrin-induced SIK1 expression

**Figure 2:**
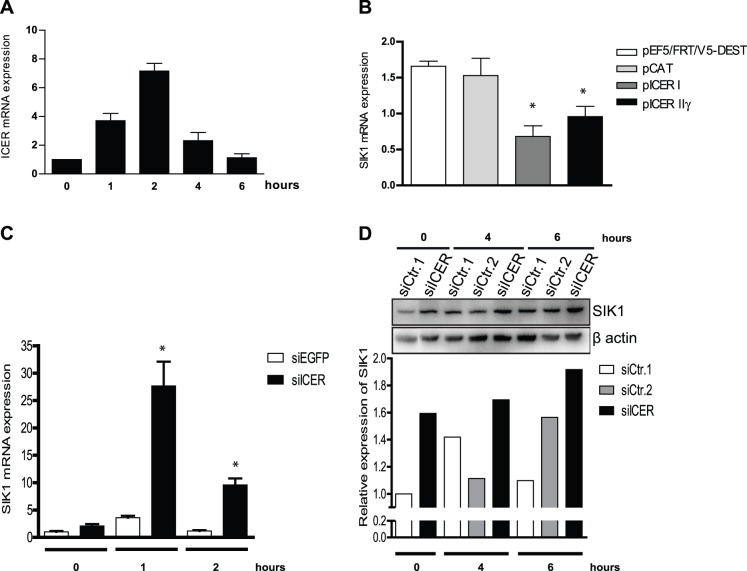
ICER represses the level of *SIK1* mRNA and protein. **A:** AGS-G_R_ cells were treated with gastrin and mRNA levels of ICER measured by qRT-PCR. Results shown are mean ± SEM of three independent biological experiments. **B:** AGS-G_R_ cells were transfected with ICER I, ICER IIγ or control expression plasmids, treated with gastrin (1 h) and mRNA levels of SIK1 measured by qRT-PCR. Results show one representative of three independent experiments; mean ± SD of three technical replicates. **C:** AGS-G_R_ cells were transfected with siRNAs, treated with gastrin and mRNA levels measured by qRT-PCR. Results show one representative of three independent experiments; mean ± SD of three technical replicates. **D:** SIK1 Western blot in cells transfected with siICER. A representative image is shown and quantified.

Our result in Figure 1A–E shows that gastrin transiently induces SIK1 mRNA and protein in gastrin responsive cells. This indicates that gastrin-induced SIK1 expression might be under negative control of a gastrin-induced repressor. Results from our genome-wide time series experiments in AR42J cells revealed that SIK1 is a primary gastrin-induced gene [4]. Treating AR42J cells with cycloheximide (CHX) (to block *de novo* protein synthesis) did not inhibit *Sik1* mRNA induction by gastrin (Figure S1B and [4]), suggesting that *SIK1* is a direct target gene of gastrin signalling. Gastrin induced higher levels of SIK1 in presence of CHX, supporting the assumption that SIK1 expression might be under negative control of a gastrin-induced repressor. We have previously shown that the transcriptional repressor ICER, which negatively regulates gene expression via binding to CRE promoter elements, is induced by gastrin [28], [29]. Since the *Sik1* promoter contains consensus CRE-binding sites [30], *SIK1* is a potential ICER target gene. We found that gastrin induces ICER expression in AGS-G_R_ cells (Figure 2A) and hypothesized that ICER might be involved in negative regulation of SIK1 expression. Ectopic expression of the ICER splice variants ICER I or ICER IIγ in AGS-G_R_ cells resulted in reduced levels of gastrin-induced SIK1 gene expression (Figure 2B). To corroborate that the repressing effect was caused by ICER, SIK1 expression was measured in presence of siRNA targeting ICER. Our results showed that siICER increases gastrin-induced SIK1 expression at the mRNA level (Figure 2C), followed by enhancement of its protein level (Figure 2D), indicating that inhibition of gastrin-induced ICER expression can de-repress SIK1 expression. Taken together, we demonstrate that ICER acts as a negative regulator of gastrin-induced SIK1 expression.

### SIK1 promote gastrin-induced transcription likely via phosphorylation of HDACs

**Figure 3:**
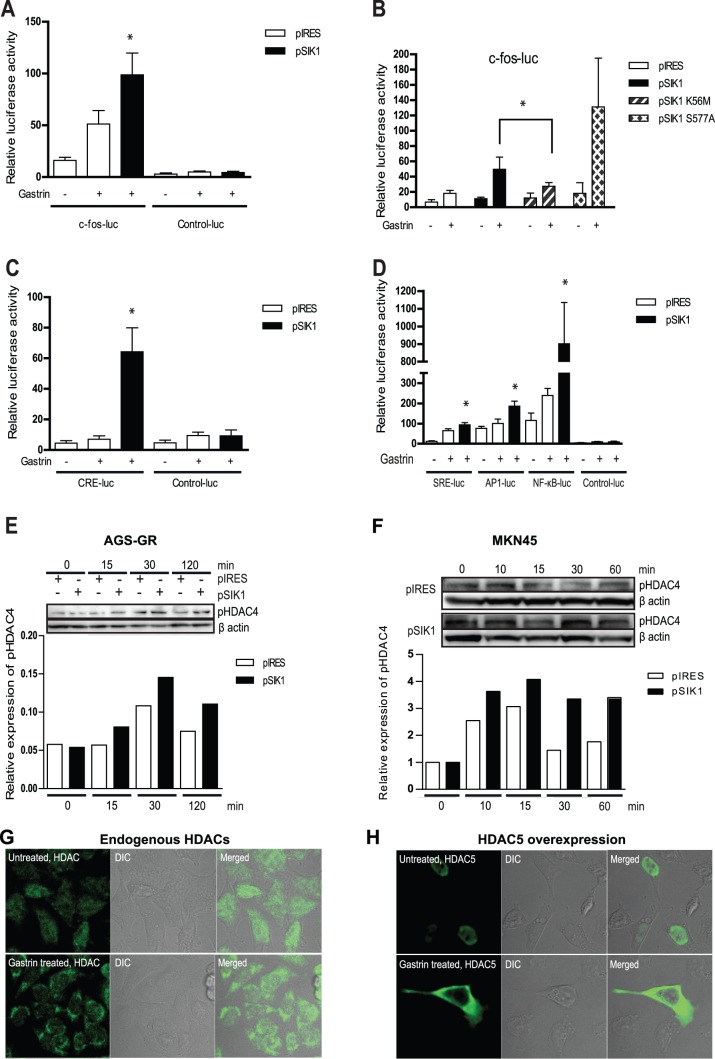
SIK1 enhances gastrin-induced transcription. AGS-G_R_ cells cotransfected with expression plasmids and reporter plasmids and treated with gastrin (4 h). Results show one representative of three independent experiments; mean ± SD of six technical replicates. **A:** Cells co-transfected with c-*fos*-luc and pIRES (control) or pSIK1. **B:** The effect of pSIK1 wt and pSIK1 mutants on c-*fos*-luc expression. **C:** Cells co-transfected with CRE-luc and pIRES (control) or pSIK1. **D:** Cells cotransfected with pSIK1 and SRE-luc, AP1-luc or NF-κB-luc. **E–F:** AGS-G_R_ cells (**E**) or MKN45 cells (**F**) transfected with pSIK1 or pIRES (control) and treated with gastrin. Phospho-HDAC4/5/7 was determined by Western blot. For both Western blots one representative of at least two independent biological experiments is shown. **G:** Intracellular localization of endogenous HDAC4/5/7. Upper panel; untreated cells, lower panel; gastrin treated cells (2 h). **H:** Intracellular localization of HDAC5 Flag. Upper panel; untreated cells, lower panel; gastrin treated cells (2 h).

Since *SIK1* is an early gastrin responsive gene, it was of interest to unravel if SIK1 was involved in downstream transcriptional activation. We and others have previously shown that c-fos is activated by gastrin [23], [31], [32], and we thus wanted to explore a possible role of SIK1 in gastrin-induced *c-fos* activation. AGS-G_R_ cells with ectopically expressed SIK1 showed a two-fold increase in gastrin-induced c-*fos-*luc transcription compared to control cells (Figure 3A).

We further determined the gastrin response in cells with ectopic expression of the mutants SIK1 K56M (kinase dead [26]) or SIK S577A (cannot be phosphorylated at Ser-577, resulting in SIK1 nuclear sequestration [27]). SIK1 K56M elicits significantly lower gastrin-induced activation of c-*fos*-luc compared to SIK1 wt (Figure 3B), supporting our anticipation that the kinase activity of SIK1 is important for the enhancing effect of gastrin-induced c-*fos* transcription. Ectopically expressed SIK1 S577A, expected to be localized exclusively in the nucleus [27], results in approximately 2.5 fold higher gastrin-induced c-*fos* activation compared to SIK1 wt (Figure 3B). Our results may indicate that SIK1 promotes gastrin-induced *c-fos* transcription by phosphorylating a nuclear target.

The c-fos promoter contains several transcription factor binding sites including CRE, SRE and AP1 [33]. To further identify promoter elements involved in SIK1-mediated transcriptional regulation, we examined the effect of ectopic SIK1 expression on gastrin-induced CRE-, SRE-, AP1- and NF-κB-driven luciferase reporter plasmids. The results show that SIK1 expression enhances gastrin-mediated transcription via CRE (Figure 3C), SRE, AP1 and NF-κB promoter elements (Figure 3D). The effect of SIK1 on gastrin-mediated transcriptional activation via this large variety of promoter elements, each of which is known to be targeted by different transcription factors, suggests that SIK1 enhances gastrin-induced transcription via a general mechanism (*e.g.* modifying chromatin) that may affect several specific promoter-transcription factor interactions.

Histone deacetylases (HDAC) are enzymes modifying histones by removing acetyl groups on the histones. Class IIa HDACs (HDAC 4/5/7/9) are recruited to specific promoters by sequence-specific DNA-binding proteins, with a subsequent deacetylation of local chromatin resulting in repression of transcription (reviewed in [34]). More recently, SIK1 was shown to phosphorylate class IIa HDACs, leading to their translocation to cytoplasm and de-repression of gene expression [30], [35], [36]. We hypothesized that phosphorylation of HDAC may represent a general mechanism underlying the enhancement of transcription that we observed in our reporter gene studies. We therefore investigated gastrin-induced HDAC4/5/7 phosphorylation in AGS-G_R_ and MKN45 cells with ectopic expression of SIK1. As shown in Figure 3E–F, gastrin induces phosphorylation of HDAC4 and overexpression of SIK1 leads to a weak but reproducible enhancement of phosphorylated HDAC4. In AGS-G_R_ cells, the SIK1-induced HDAC4 phosphorylation is observable already after 15 min of gastrin-stimulation and is still markedly enhanced after 2 h. In MKN45 cells the gastrin induced phosphorylation of HDAC4 was observed from 10 to 60 min (Figure 3F). We also show that gastrin treatment induces nuclear export of endogenous HDACs (HDAC4/5/7) and ectopic HDAC5 in AGS-G_R_ cells (Figure 3G–H). Our results suggest that the observed enhancement of gastrin-induced transcription can be due to a mechanism whereby the promoters are rendered more receptive to activating transcription mechanisms after SIK1-mediated nuclear export of phosphorylated HDACs.

### SIK1 represses gastrin-induced migration of AGS-G_R_ cells

**Figure 4:**
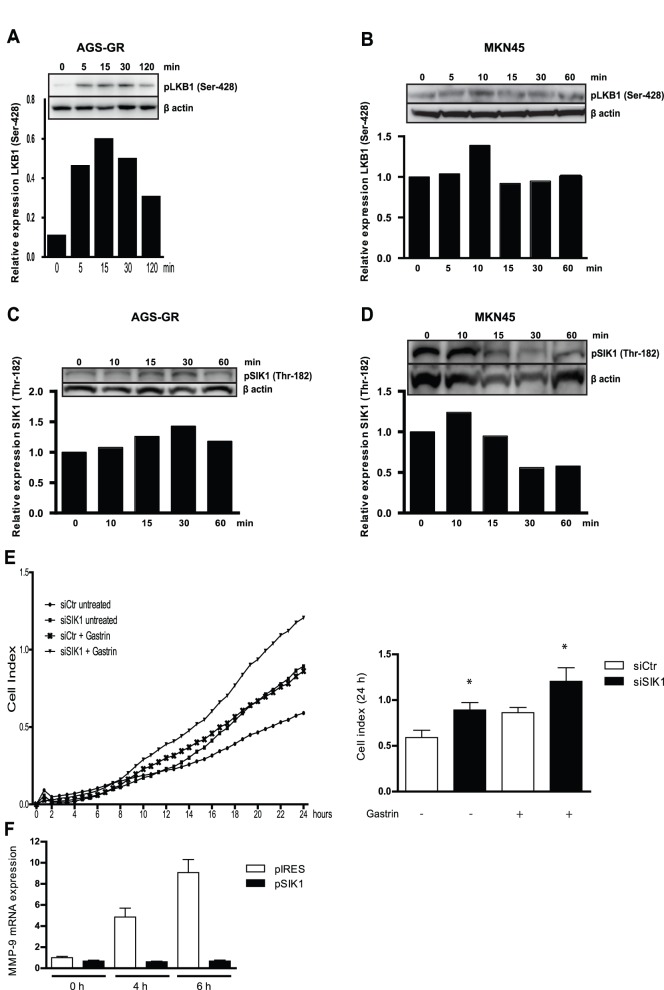
SIK1 inhibits migration in AGS-G_R_ cells via suppression of MMP-9. **A–B:** AGS-G_R_ cells (**A**) and MKN45 cells (**B**) were treated with gastrin, and phospho-LKB1 (Ser-428) protein levels determined by Western blot. The phospho-LKB1 bands from a representative experiment are shown. **C–D:** AGS-G_R_ cells (**C**) and MKN45 (**D**) were treated with gastrin, and phospho-SIK1 (Thr-182) protein levels determined by Western blot. The phospho-SIK1 bands from a representative experiment are shown. **E:** AGS-G_R_ cells transfected with siSIK1 or siCtr and real-time cell migration monitored (0–24 h). Results show one representative of three independent experiments (mean ±SD of three technical replicates). **F:** MMP-9 mRNA expression in cells transfected with pSIK1 and treated with gastrin. Results show one representative of three independent experiments, (mean ± SD).

SIK1 activity is promoted by phosphorylation at Thr-182 implemented by upstream kinases like LKB1 [37]. Cheng and co-workers [16] have shown that LKB1-mediated activation of SIK1 suppresses metastasis and invasion. To establish a link between gastrin, LKB1 and SIK1, the phosphorylation status of LKB1 and SIK1 was examined in protein lysates from gastrin-treated AGS-G_R_ and MKN45 cells. Figure 4A–B shows that gastrin induces phosphorylation of LKB1 at Ser-428, an amino acid residue shown to be involved in increased apoptosis in endothelial cells [38]. We also find that gastrin mediates SIK (Thr-182) phosphorylation in both cell lines (Figure 4C–D). Taken together, this suggests that gastrin-induced LKB1 phosphorylation is responsible for the subsequent SIK1 activation.

In cancer biology SIK1 expression has been associated with a tumour suppressor function [13], [14], [15], [16]. Cell migration is an important feature of oncogenesis, and gastrin is shown to induce migration in AGS-G_R_ cells [5]. Thus we proceeded to examine the role of SIK1 in gastrin-induced migration. AGS-G_R_ cells, which have the ability to migrate *in vitro* [39] were transfected with siSIK1+/− gastrin and migration assessed using real-time cell monitoring assay (xCELLigence technology). We show that cells with SIK1 knockdown display significantly higher migratory activity compared to control cells (transfected with Silencer Negative Control siRNA [siCtr]), and that this difference is enhanced in gastrin-treated cells (Figure 4E), suggesting that SIK1 participates in migration-repressing processes in gastric cancer cells.

Several members of the matrix metalloproteinase (MMP) family are shown to trigger cancer cell migration, so we hypothesized that the inhibitory effect of SIK1 on migration may involve regulation of MMPs. Since MMP-9 is one of several extracellular proteins reported to participate in gastrin-mediated regulation of invasion [6], we hypothesized that the inhibitory effect of SIK1 on migration may involve regulation of MMP-9. AGS-G_R_ cells were transfected with pSIK1 and treated with gastrin (0–6 h). We found that ectopically expressed SIK1 reduced the gastrin induced *MMP-9* expression (Figure 4F). Our results suggest that the SIK1-mediated reduction of gastrin-induced migration could in part be explained by reduced levels of the migration enhancing protein MMP-9.

## Discussion

Several studies have reported that α- and β-adrenergic receptors and Ser/Thr kinase receptors mediate regulation of SIK1 [27], [40], [41], [42]. In the present study we show that gastrin-mediated signalling, known to involve the Gq/G_11_-coupled CCK2 receptor and signalling pathways like PI3K-PKB/Akt, PKC and MEK-ERK1/2 [29], [31], [43], induces transient SIK1 expression in adenocarcinoma cells. Gastrin treatment did not give any evident translocation of ectopically expressed SIK1 proteins nor endogenous CRTC2, suggesting that the well-established SIK1/CRTC2/CREB axis [25] does not occur or play any prominent role in gastrin mediated signalling.

We identified SIK1 as a primary gastrin-responsive gene, negatively regulated by the transcriptional repressor ICER. We have previously shown that ICER is involved in down-regulation of gastrin-induced genes like *Cyclin D1* and *c-fos* [23]. The observations in the current study thus support the hypothesis that induction of ICER is part of an intracellular feedback mechanism contributing to the attenuation of biological responses to gastrin.

**Figure 5:**
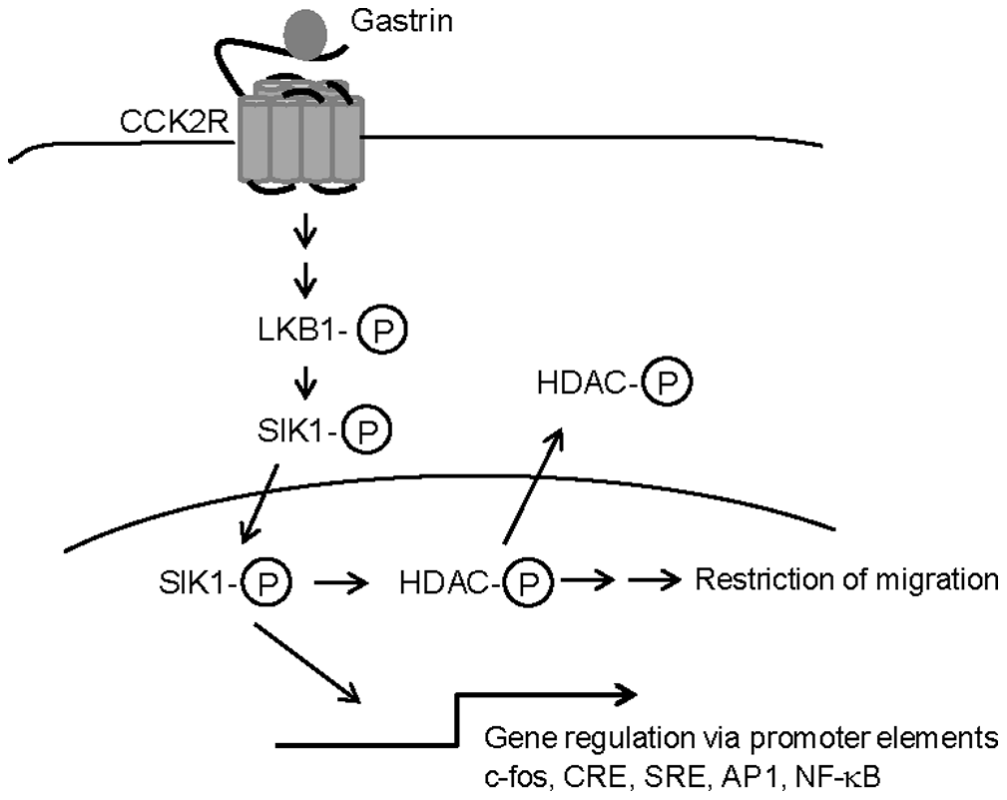
The role of SIK1 in gastrin responsive cells. Gastrin binds to the CCK2 receptor (CCK2R) and activates the LKB1–SIK1 signalling pathway in adenocarcinoma cells. SIK1 mediated phosphorylation of HDAC leads to cytosolic translocation and activation of transcription. In the gastric adenocarcinoma cell line AGS-G_R_ ectopic SIK1 inhibits migration.

We demonstrate that ectopically expressed SIK1 enhances gastrin-induced phosphorylation of HDAC4 (illustrated in Figure 5), confirming the role of SIK1 as a class IIa HDAC kinase, as described in other cell systems [30], [35], [44]. However, the role of gastrin in mediating HDAC phosphorylation and export has not been shown earlier. The class IIa HDACs (HDAC4/5/7/9) are thought to act as transcriptional corepressors by deacetylating nucleosomal histones. Class IIa HDAC-mediated repression is relieved by phosphorylation which results in their cytoplasmic accumulation, sequestering them from histone substrates and renders them enzymatically inactive (reviewed in [34]). SIK1 phosphorylation of HDAC4 might be the mechanism underlying our observed SIK1-mediated enhancement of gastrin-induced transcription. This coincides with the findings of Pan *et al* [45] who reported that HDAC4 represses *c-fos* transcription, and that phosphorylation of HDAC4 will de-repress the transcription [45]. Others have shown that the CREB/ATF binding site in the mouse *c-fos* promoter is subject to transcriptional repression via chromatin remodelling mechanisms [46], [47]. Hence, we propose that SIK1, upon gastrin stimulation, mediates phosphorylation of HDACs, induces nuclear export and thereby relieving HDAC-induced repression of *c-fos* transcription. Since our results included SIK1 elicited enhancement of transcription via a variety of promoter elements (CRE, SRE, AP1, NF-κB), we suggest that SIK1-mediated phosphorylations and subsequent inactivation of HDACs may enhance the gastrin-mediated transcription of a large number of genes, as also reported by others [30], [44].

We show that SIK1 knockdown by siRNA brings along higher migratory activity in gastric adenocarcinoma cells, indicating that SIK1 suppresses gastrin-mediated migration. Our observation that LKB1 Ser-428 and SIK1 Thr-182 are phosphorylated upon gastrin treatment, suggests that gastrin activates SIK1 via LKB1 in AGS-G_R_ and MKN45 cells. Ectopically expressed LKB1 is also reported to reduce MMP-9 mRNA and protein expression, and to inhibit invasion of breast cancer cells, but the molecular mechanisms involved have not been explored [48], [49]. Our experiments suggest that the reduction in migration involves MMP-9, which lead us to speculate that SIK1 affects HDAC activity resulting in decreased transcription of MMP-9. This could be by transcriptional activation of a MMP-9 repressor. Alternatively, SIK1 may inactivate a HDAC involved in activation of MMP-9 gene expression, analogous to the role of HDAC7 in transcriptional activation of hypoxia-inducible factor 1α (HIF-1α) [50]. The class IIa HDAC7 is shown to increase transcriptional activity of HIF-1α through the formation of a complex with HIF-1α and p300 [50]. Both of these mechanisms will result in lower transcriptional activation of MMP-9. Previously MMP-9 was demonstrated to be up-regulated in gastric biopsies of the majority of patients with elevated plasma gastrin compared to healthy controls [6], and Wroblewski *et al* [6] speculate that gastrin may be one of the factors regulating MMP-9 expression and activation in the gastric epithelium. The recent finding demonstrating that gastrin may change the epigenetic status of a promoter, including the histone acetylation of local chromatin [51], leads us to speculate that this may be mediated via SIK1-induced regulation of class IIa HDACs resulting in changes in histone modification and chromatin remodelling. However, further experiments are needed to unfold such possible mechanisms.

To conclude, in this study we characterize a function of SIK1 in gastric adenocarcinoma cells. We show that SIK1 is regulated by gastrin and that SIK1 inhibits gastrin induced migration.

## Supporting Information

Figure S1 A: SIK1 Western blot of gastrin treated AGS-G_R_ cells. A representative image is shown. The SIK1 bands from the experiment are quantified. B: Microarray gene expression analysis showing the effect of the protein synthesis inhibitor cycloheximide (CHX) on gastrin-induced SIK1 mRNA expression in AR42J cells. Cells were pre-treated with CHX (10 µg/ml) for 30 min before gastrin (10 nM) were added. Expression level relative to time point zero is shown.
(EPS)
